# Impeachment of Professor Hall, of Baltimore

**Published:** 1844-03

**Authors:** 


### IMPEACHMENT OF PROFESSOR HALL, OF BALTIMORE.

We learn, from the Boston Medical and Surgical Journal, of the 28th of February, that the difficulty among the medical professors of the University of Maryland, last season, led to an impeachment of Professor Hall. He was charged with not properly sustaining the chair of which he had been so long the incumbent, with an unwillingness to pay old scores, &c. A very stout pamphlet, it seems, has been published by a committee appointed by the Regents, setting forth the testimony "in the Matter of the Impeachment." And the result of the whole matter is, that Professor Hall stands his ground "as popular as ever, and as obnoxions too." The charges, according to the evidence, were not sustained. The class in the University is reported to have been larger, last winter, than usual.

